# 4,4′-Dimethyl-2,2′-[(3-aza­pentane-1,5-di­yl)bis­(nitrilo­methyl­idyne)]diphenol

**DOI:** 10.1107/S1600536808018837

**Published:** 2008-06-28

**Authors:** Fang-Fang Dang, Xin-Wei Wang, Qing-Cui Yang, Guo-Ping Han

**Affiliations:** a School of Science, Xi’an University of Architecture and Technology, Xi’an 710055, People’s Republic of China; b Xi’an LiBang Pharmaceutical Co. Ltd, Xi’an 710086, People’s Republic of China

## Abstract

In the crystal structure of the title Schiff base, C_20_H_25_N_3_O_2_, the salicylaldimine groups at each end of the mol­ecule are essentially planar and make a dihedral angle of 84.94 (3)° with each other. There are strong intra­molecular O—H⋯N hydrogen bonds and a weak inter­molecular N—H⋯O hydrogen bond.

## Related literature

For related literature, see: Rodriguez de Barbarin *et al.* (1994 ▶).
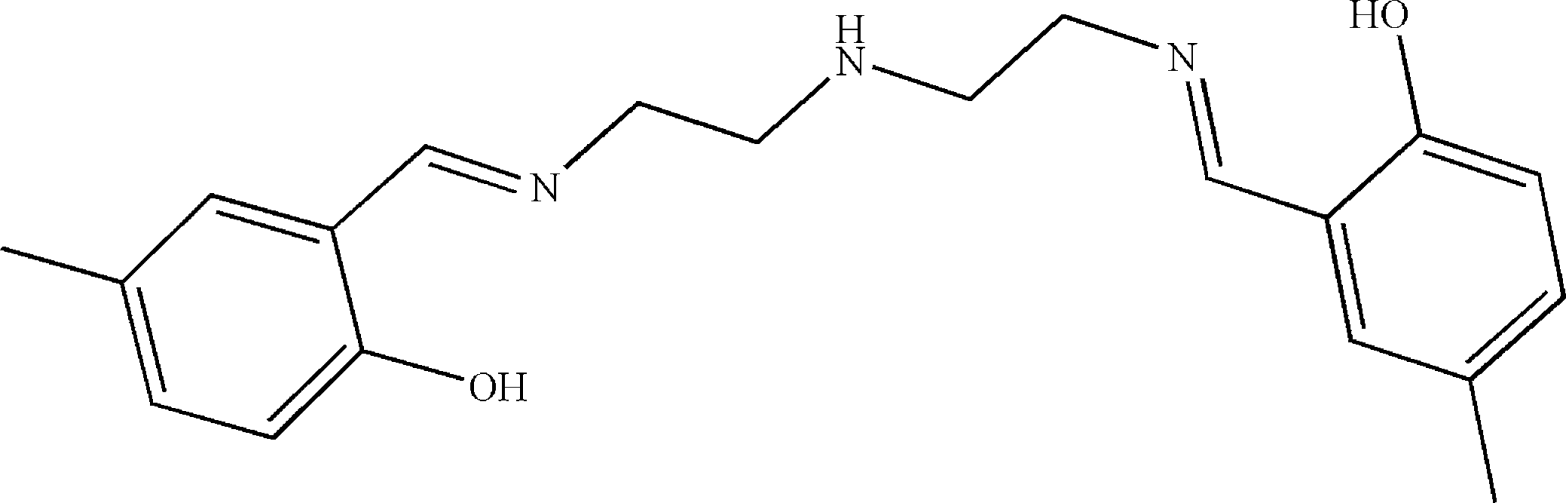


## Experimental

### 

#### Crystal data

C_20_H_25_N_3_O_2_
*M* *_r_* = 339.43
Orthorhombic, 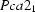

*a* = 9.132 (4) Å
*b* = 5.834 (3) Å
*c* = 34.365 (16) Å
*V* = 1830.8 (15) Å^3^
*Z* = 4
Mo *K*α radiation
μ = 0.08 mm^−1^
*T* = 296 (2) K
0.34 × 0.32 × 0.28 mm

#### Data collection

Bruker APEXII area-detector diffractometer
Absorption correction: multi-scan (**SADABS**; Sheldrick, 1996 ▶) *T*_min_ = 0.970, *T*_max_ = 0.981
14593 measured reflections
2125 independent reflections
1867 reflections with *I* > 2σ(*I*)
*R*_int_ = 0.031

#### Refinement

*R*[*F* ^2^ > 2σ(*F* ^2^)] = 0.037
*wR*(*F* ^2^) = 0.108
*S* = 1.07
2125 reflections
228 parameters
1 restraint
H-atom parameters constrained
Δρ_max_ = 0.15 e Å^−3^
Δρ_min_ = −0.18 e Å^−3^

### 

Data collection: *APEX2* (Bruker, 2004 ▶); cell refinement: *SAINT* (Bruker, 2004 ▶); data reduction: *SAINT*; program(s) used to solve structure: *SHELXS97* (Sheldrick, 2008 ▶); program(s) used to refine structure: *SHELXL97* (Sheldrick, 2008 ▶); molecular graphics: *SHELXTL* (Sheldrick, 2008 ▶); software used to prepare material for publication: *SHELXTL*.

## Supplementary Material

Crystal structure: contains datablocks global, I. DOI: 10.1107/S1600536808018837/is2306sup1.cif

Structure factors: contains datablocks I. DOI: 10.1107/S1600536808018837/is2306Isup2.hkl

Additional supplementary materials:  crystallographic information; 3D view; checkCIF report

## Figures and Tables

**Table 1:** Hydrogen-bond geometry (Å, °)

*D*—H⋯*A*	*D*—H	H⋯*A*	*D*⋯*A*	*D*—H⋯*A*
O1—H1*A*⋯N1	0.82	1.89	2.614 (4)	146
O2—H2*A*⋯N2	0.82	1.88	2.602 (3)	146
N3—H3*A*⋯O1^i^	0.86	2.54	3.140 (3)	128

Symmetry code: (i) 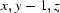.

**Fig. 1.**
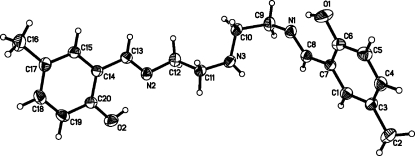
The structure of (I), showing 30% probability displacement ellipsoids and the atom-numbering scheme.

## supplementary crystallographic information

### Comment

It was reported that zinc coordinated by phenolate groups in a Schiff-base ligand can act as a nucleophile to catalyze ester hydrolysis (Rodriguez de Barbarin *et al.*, 1994). These results promoted us to investigate linear amine-phenol ligands obtained by reducing Schiff bases, which have greater flexibility, better water solubility and more inertness to hydrolytic decomposition than corresponding Schiff bases. While a number of their complexes with transition metals and main group metals have been reported, the crystal structures of these Schiff-base ligands remain relatively unexplored. So we present here the crystal structure of the title compound, *N*,*N*'-bis(5-methylsalicylidene)-1,5-diamino-3-azapentane, (I).

The molecular structure of (I) is illustrated in Fig. 1. Compound (I) is a typical salicylaldehyde schiff derivative with normal geometric parameters. The two pendant moieties attached to the ends of the C—C—N—C—C backbone adopt a *cis* conformation. The N3 atom exhibits tetrahedral *sp*^3^ hybridization, whereas the two amide N atoms display planar *sp*^2^ hybridization. The C8—N1 and C13—N2 bonds show the expected double-bond character. In our case, the salicylaldimine moiety is nearly planar. The dihedral angle between the salicylaldimine groups is 84.94 (3)°. The crystal structure of (I) is stabilized by intramolecular O—H···N hydrogen bonds and an intermolecular N—H···O hydrogen bond (Table 1).

### Experimental

*N*-(2-aminoethyl)ethane-1,2-diamine (0.01 mol, 1.03 g) and 2-hydroxy-5-methylbenzaldehyde (0.02 mol, 2.72 g) were dissolved in ethanol and the solution was refluxed for 1 h. After evaporation, a crude product was recrystallized twice from ethanol to give a pure yellow product. Yield: 80.5%. Calcd. for C_20_H_25_N_3_O_2_: C 70.77, H 7.42, N 12.38; Found: C 71.02, H 7.47, N 12.27%.

### Refinement

All H atoms were located from a difference Fourier map. Then H atoms were placed in geometrically idealized positions (C—H = 0.93–0.97 Å, O—H = 0.82 Å and N—H = 0.86 Å) and constrained to ride on their parent atoms, with *U*_iso_(H) = 1.2*U*_eq_(C, N) or 1.5*U*_eq_(O). In the absence of significant anomalous scattering effects, Friedel pairs have been merged.

### Crystal data

**Table:**

C_20_H_25_N_3_O_2_	*F*_000_ = 728
*M**_r_* = 339.43	*D*_x_ = 1.231 Mg m^−^^3^
Orthorhombic, *P**c**a*2_1_	Mo *K*α radiation λ = 0.71073 Å
Hall symbol: P 2c -2ac	Cell parameters from 2105 reflections
*a* = 9.132 (4) Å	θ = 1.0–27.5º
*b* = 5.834 (3) Å	µ = 0.08 mm^−^^1^
*c* = 34.365 (16) Å	*T* = 296 (2) K
*V* = 1830.8 (15) Å^3^	Block, yellow
*Z* = 4	0.34 × 0.32 × 0.28 mm

### Data collection

**Table:**

Bruker APEXII area-detector diffractometer	2125 independent reflections
Radiation source: fine-focus sealed tube	1867 reflections with *I* > 2σ(*I*)
Monochromator: graphite	*R*_int_ = 0.031
*T* = 296(2) K	θ_max_ = 27.5º
φ and ω scans	θ_min_ = 2.4º
Absorption correction: multi-scan(*SADABS*; Sheldrick, 1996)	*h* = −11→11
*T*_min_ = 0.970, *T*_max_ = 0.981	*k* = −7→7
14593 measured reflections	*l* = −44→44

### Refinement

**Table:**

Refinement on *F*^2^	Secondary atom site location: difference Fourier map
Least-squares matrix: full	Hydrogen site location: inferred from neighbouring sites
*R*[*F*^2^ > 2σ(*F*^2^)] = 0.037	H-atom parameters constrained
*wR*(*F*^2^) = 0.108	*w* = 1/[σ^2^(*F*_o_^2^) + (0.0588*P*)^2^ + 0.2052*P*] where *P* = (*F*_o_^2^ + 2*F*_c_^2^)/3
*S* = 1.07	(Δ/σ)_max_ < 0.001
2125 reflections	Δρ_max_ = 0.15 e Å^−^^3^
228 parameters	Δρ_min_ = −0.18 e Å^−^^3^
1 restraint	Extinction correction: none
Primary atom site location: structure-invariant direct methods	

### Special details

**Table:**

Geometry. All e.s.d.'s (except the e.s.d. in the dihedral angle between two l.s. planes) are estimated using the full covariance matrix. The cell e.s.d.'s are taken into account individually in the estimation of e.s.d.'s in distances, angles and torsion angles; correlations between e.s.d.'s in cell parameters are only used when they are defined by crystal symmetry. An approximate (isotropic) treatment of cell e.s.d.'s is used for estimating e.s.d.'s involving l.s. planes.
Refinement. Refinement of *F*^2^ against ALL reflections. The weighted *R*-factor *wR* and goodness of fit *S* are based on *F*^2^, conventional *R*-factors *R* are based on *F*, with *F* set to zero for negative *F*^2^. The threshold expression of *F*^2^ > σ(*F*^2^) is used only for calculating *R*-factors(gt) *etc*. and is not relevant to the choice of reflections for refinement. *R*-factors based on *F*^2^ are statistically about twice as large as those based on *F*, and *R*- factors based on ALL data will be even larger.

### Fractional atomic coordinates and isotropic or equivalent isotropic displacement parameters (Å^2^)

**Table:**

	*x*	*y*	*z*	*U*_iso_*/*U*_eq_	
O1	0.8321 (3)	1.4932 (4)	0.36346 (6)	0.0766 (7)	
H1A	0.8862	1.4181	0.3493	0.115*	
O2	0.7661 (2)	0.0213 (3)	0.18913 (5)	0.0594 (5)	
H2A	0.8305	0.0960	0.1999	0.089*	
N1	1.0105 (2)	1.1600 (4)	0.34511 (6)	0.0506 (5)	
N2	0.9202 (2)	0.3855 (3)	0.20445 (5)	0.0479 (4)	
N3	1.0062 (2)	0.7202 (3)	0.29474 (6)	0.0520 (5)	
H3A	1.0210	0.6583	0.3171	0.062*	
C1	0.8251 (3)	1.0370 (4)	0.43648 (7)	0.0455 (5)	
H1C	0.8686	0.8960	0.4416	0.055*	
C2	0.6782 (4)	0.9888 (6)	0.49830 (9)	0.0762 (9)	
H2B	0.7544	0.8830	0.5051	0.114*	
H2C	0.5895	0.9059	0.4931	0.114*	
H2D	0.6622	1.0933	0.5195	0.114*	
C3	0.7226 (3)	1.1209 (4)	0.46259 (7)	0.0495 (6)	
C4	0.6605 (3)	1.3338 (5)	0.45429 (7)	0.0545 (6)	
H4A	0.5923	1.3947	0.4715	0.065*	
C5	0.6967 (3)	1.4566 (4)	0.42153 (8)	0.0563 (6)	
H5A	0.6526	1.5977	0.4170	0.068*	
C6	0.7992 (3)	1.3710 (4)	0.39506 (7)	0.0487 (5)	
C7	0.8657 (2)	1.1551 (4)	0.40291 (6)	0.0399 (5)	
C8	0.9752 (2)	1.0605 (4)	0.37676 (7)	0.0437 (5)	
H8A	1.0208	0.9233	0.3833	0.052*	
C9	1.1263 (3)	1.0645 (5)	0.32037 (7)	0.0584 (7)	
H9A	1.1836	0.9560	0.3354	0.070*	
H9B	1.1911	1.1871	0.3122	0.070*	
C10	1.0648 (3)	0.9444 (4)	0.28455 (7)	0.0532 (6)	
H10A	0.9879	1.0376	0.2732	0.064*	
H10B	1.1416	0.9266	0.2653	0.064*	
C11	0.9210 (3)	0.6085 (4)	0.26450 (6)	0.0472 (5)	
H11A	0.8477	0.7152	0.2550	0.057*	
H11B	0.8698	0.4788	0.2758	0.057*	
C12	1.0123 (3)	0.5243 (5)	0.23003 (7)	0.0521 (6)	
H12A	1.0510	0.6543	0.2158	0.063*	
H12B	1.0940	0.4335	0.2394	0.063*	
C13	0.8784 (3)	0.4705 (4)	0.17204 (7)	0.0441 (5)	
H13A	0.9125	0.6143	0.1647	0.053*	
C14	0.7784 (2)	0.3473 (3)	0.14626 (6)	0.0412 (5)	
C15	0.7306 (3)	0.4500 (4)	0.11167 (7)	0.0466 (5)	
H15A	0.7683	0.5925	0.1048	0.056*	
C16	0.5766 (4)	0.4651 (6)	0.05050 (9)	0.0746 (8)	
H16A	0.4734	0.4399	0.0474	0.112*	
H16B	0.6279	0.4036	0.0285	0.112*	
H16C	0.5955	0.6266	0.0524	0.112*	
C17	0.6291 (3)	0.3464 (4)	0.08737 (7)	0.0519 (6)	
C18	0.5756 (3)	0.1310 (4)	0.09836 (8)	0.0562 (6)	
H18A	0.5074	0.0582	0.0825	0.067*	
C19	0.6215 (3)	0.0237 (4)	0.13212 (8)	0.0534 (6)	
H19A	0.5838	−0.1193	0.1387	0.064*	
C20	0.7242 (3)	0.1288 (4)	0.15635 (7)	0.0448 (5)	

### Atomic displacement parameters (Å^2^)

**Table:**

	*U*^11^	*U*^22^	*U*^33^	*U*^12^	*U*^13^	*U*^23^
O1	0.0892 (16)	0.0680 (11)	0.0725 (13)	0.0077 (11)	0.0161 (12)	0.0338 (10)
O2	0.0733 (12)	0.0477 (9)	0.0574 (10)	0.0009 (8)	0.0025 (9)	0.0103 (8)
N1	0.0448 (10)	0.0629 (12)	0.0440 (10)	−0.0085 (9)	0.0049 (8)	−0.0061 (9)
N2	0.0467 (10)	0.0535 (11)	0.0434 (10)	0.0017 (9)	0.0034 (9)	−0.0054 (9)
N3	0.0551 (11)	0.0628 (12)	0.0381 (9)	−0.0069 (10)	−0.0017 (9)	0.0057 (9)
C1	0.0484 (13)	0.0446 (11)	0.0435 (12)	0.0005 (9)	−0.0054 (10)	0.0057 (9)
C2	0.091 (2)	0.085 (2)	0.0526 (15)	−0.0057 (18)	0.0175 (17)	0.0134 (15)
C3	0.0516 (14)	0.0570 (13)	0.0400 (12)	−0.0081 (11)	0.0041 (10)	0.0013 (10)
C4	0.0518 (14)	0.0562 (14)	0.0554 (14)	−0.0016 (11)	0.0111 (11)	−0.0116 (11)
C5	0.0554 (15)	0.0471 (12)	0.0664 (16)	0.0058 (11)	0.0051 (13)	0.0010 (11)
C6	0.0491 (13)	0.0460 (12)	0.0511 (12)	−0.0032 (10)	−0.0004 (10)	0.0066 (10)
C7	0.0378 (11)	0.0429 (10)	0.0391 (11)	−0.0038 (8)	−0.0007 (9)	−0.0001 (8)
C8	0.0377 (11)	0.0502 (12)	0.0432 (11)	−0.0037 (9)	−0.0034 (9)	−0.0039 (9)
C9	0.0437 (13)	0.0815 (17)	0.0501 (13)	−0.0115 (13)	0.0066 (11)	−0.0145 (13)
C10	0.0551 (14)	0.0640 (14)	0.0405 (12)	−0.0100 (11)	0.0078 (11)	−0.0025 (11)
C11	0.0450 (12)	0.0540 (12)	0.0427 (12)	−0.0070 (10)	0.0053 (10)	−0.0001 (10)
C12	0.0410 (11)	0.0667 (14)	0.0487 (13)	0.0014 (11)	0.0006 (10)	−0.0066 (11)
C13	0.0424 (11)	0.0437 (11)	0.0463 (12)	−0.0010 (9)	0.0062 (9)	−0.0030 (9)
C14	0.0428 (11)	0.0384 (10)	0.0422 (11)	0.0013 (9)	0.0085 (9)	−0.0041 (9)
C15	0.0522 (13)	0.0417 (11)	0.0460 (12)	−0.0025 (10)	0.0059 (10)	−0.0008 (9)
C16	0.089 (2)	0.083 (2)	0.0516 (15)	−0.0001 (18)	−0.0152 (15)	0.0008 (14)
C17	0.0567 (15)	0.0551 (13)	0.0438 (12)	0.0038 (11)	0.0019 (11)	−0.0057 (10)
C18	0.0580 (15)	0.0545 (14)	0.0559 (15)	−0.0049 (11)	0.0013 (12)	−0.0167 (12)
C19	0.0575 (15)	0.0396 (11)	0.0632 (15)	−0.0071 (10)	0.0107 (12)	−0.0086 (11)
C20	0.0494 (12)	0.0386 (11)	0.0463 (12)	0.0021 (9)	0.0108 (10)	−0.0029 (9)

### Geometric parameters (Å, °)

**Table:**

O1—C6	1.333 (3)	C9—H9A	0.9700
O1—H1A	0.8200	C9—H9B	0.9700
O2—C20	1.345 (3)	C10—H10A	0.9700
O2—H2A	0.8200	C10—H10B	0.9700
N1—C8	1.274 (3)	C11—C12	1.530 (3)
N1—C9	1.467 (3)	C11—H11A	0.9700
N2—C13	1.277 (3)	C11—H11B	0.9700
N2—C12	1.461 (3)	C12—H12A	0.9700
N3—C11	1.453 (3)	C12—H12B	0.9700
N3—C10	1.456 (3)	C13—C14	1.461 (3)
N3—H3A	0.8600	C13—H13A	0.9300
C1—C3	1.386 (3)	C8—H8A	0.9300
C1—C7	1.394 (3)	C14—C15	1.401 (3)
C1—H1C	0.9300	C14—C20	1.411 (3)
C2—C3	1.505 (4)	C15—C17	1.386 (3)
C2—H2B	0.9600	C15—H15A	0.9300
C2—H2C	0.9600	C16—C17	1.521 (4)
C2—H2D	0.9600	C16—H16A	0.9600
C3—C4	1.395 (4)	C16—H16B	0.9600
C4—C5	1.375 (4)	C16—H16C	0.9600
C4—H4A	0.9300	C17—C18	1.400 (4)
C5—C6	1.397 (3)	C18—C19	1.383 (4)
C5—H5A	0.9300	C18—H18A	0.9300
C6—C7	1.424 (3)	C19—C20	1.396 (3)
C7—C8	1.453 (3)	C19—H19A	0.9300
C9—C10	1.524 (3)		
			
C6—O1—H1A	109.5	N3—C11—C12	113.95 (19)
C20—O2—H2A	109.5	N3—C11—H11A	108.8
C8—N1—C9	120.3 (2)	C12—C11—H11A	108.8
C13—N2—C12	118.8 (2)	N3—C11—H11B	108.8
C11—N3—C10	115.3 (2)	C12—C11—H11B	108.8
C11—N3—H3A	122.3	H11A—C11—H11B	107.7
C10—N3—H3A	122.3	N2—C12—C11	109.28 (19)
C3—C1—C7	122.7 (2)	N2—C12—H12A	109.8
C3—C1—H1C	118.6	C11—C12—H12A	109.8
C7—C1—H1C	118.6	N2—C12—H12B	109.8
C3—C2—H2B	109.5	C11—C12—H12B	109.8
C3—C2—H2C	109.5	H12A—C12—H12B	108.3
H2B—C2—H2C	109.5	N2—C13—C14	121.6 (2)
C3—C2—H2D	109.5	N2—C13—H13A	119.2
H2B—C2—H2D	109.5	C14—C13—H13A	119.2
H2C—C2—H2D	109.5	N1—C8—C7	121.9 (2)
C1—C3—C4	117.2 (2)	N1—C8—H8A	119.0
C1—C3—C2	122.0 (2)	C7—C8—H8A	119.0
C4—C3—C2	120.9 (2)	C15—C14—C20	119.0 (2)
C5—C4—C3	122.3 (2)	C15—C14—C13	119.9 (2)
C5—C4—H4A	118.9	C20—C14—C13	121.0 (2)
C3—C4—H4A	118.9	C17—C15—C14	122.2 (2)
C4—C5—C6	120.5 (2)	C17—C15—H15A	118.9
C4—C5—H5A	119.7	C14—C15—H15A	118.9
C6—C5—H5A	119.7	C17—C16—H16A	109.5
O1—C6—C5	119.4 (2)	C17—C16—H16B	109.5
O1—C6—C7	122.0 (2)	H16A—C16—H16B	109.5
C5—C6—C7	118.6 (2)	C17—C16—H16C	109.5
C1—C7—C6	118.7 (2)	H16A—C16—H16C	109.5
C1—C7—C8	120.4 (2)	H16B—C16—H16C	109.5
C6—C7—C8	120.8 (2)	C15—C17—C18	117.5 (2)
N1—C9—C10	112.2 (2)	C15—C17—C16	120.9 (2)
N1—C9—H9A	109.2	C18—C17—C16	121.5 (3)
C10—C9—H9A	109.2	C19—C18—C17	121.8 (2)
N1—C9—H9B	109.2	C19—C18—H18A	119.1
C10—C9—H9B	109.2	C17—C18—H18A	119.1
H9A—C9—H9B	107.9	C18—C19—C20	120.3 (2)
N3—C10—C9	110.8 (2)	C18—C19—H19A	119.8
N3—C10—H10A	109.5	C20—C19—H19A	119.8
C9—C10—H10A	109.5	O2—C20—C19	119.1 (2)
N3—C10—H10B	109.5	O2—C20—C14	121.8 (2)
C9—C10—H10B	109.5	C19—C20—C14	119.1 (2)
H10A—C10—H10B	108.1		

### Hydrogen-bond geometry (Å, °)

**Table:**

*D*—H···*A*	*D*—H	H···*A*	*D*···*A*	*D*—H···*A*
O1—H1A···N1	0.82	1.89	2.614 (4)	146
O2—H2A···N2	0.82	1.88	2.602 (3)	146
N3—H3A···O1^i^	0.86	2.54	3.140 (3)	128

Symmetry codes:  (i) *x*, *y*−1, *z*.
